# Cyclooxygenase expression is not required for release of arachidonic acid from cells by some nonsteroidal anti-inflammatory drugs and cancer preventive agents

**DOI:** 10.1186/1471-2210-6-7

**Published:** 2006-03-29

**Authors:** Lawrence Levine

**Affiliations:** 1 Department of Biochemistry, Brandeis University, Waltham, MA 02454, USA

## Abstract

**Background:**

Nonsteroidal anti-inflammatory drugs (NSAIDs) have been shown to be effective in inhibiting colorectal cancer. Cyclooxygenase activity is thought to mediate, in part, this cancer preventive effect. From observations made when cells that express cyclooxygenase activity were treated with NSAIDs and known cancer preventive agents, I have postulated that arachidonic acid (AA) release is associated with cancer prevention. In this study, the effects of NSAIDs on two cells that do not express cycloxygenase activity are detailed.

**Results:**

NSAIDs and several cancer preventive agents release AA from human colon cancer cells (the HCT-15 cell line). The concentrations of NSAIDs required to release significant amounts of AA from the HCT-15 cells are greater than those required to inhibit the lactacystin plus 12-0-tetradecanoyl-13-acetate stimulated cyclooxygenase activity of rat liver cells. NSAIDs, tamoxifen and simvastatin were found to hemolyze erythrocyte cells which also do not express cyclooxygenase activity

**Conclusion:**

The data demonstrate that AA release is independent of cyclooxygenase activity and together with hemolysis suggest that intercalation of the plasma membrane by some NSAIDs and cancer preventive agents, e.g. tamoxifen, mediates this release. A mechanism by which many of these drugs affect several diverse biologic properties including deesterification of membrane phospholipids by phospholipases to release AA is presented.

## Background

Nonsteroidal anti-inflammatory drugs (NSAIDs) inhibit cyclooxygenase (COX) activity and the local production of COX products that affect inflammation [1]. Nevertheless, the role of NSAIDs in several biologic pathways, at high doses, has been questioned [2,3]. Some NSAIDs have been shown to be cancer preventive [4,5]. The role of COX in cancer prevention is suggested by the findings that COX is overexpressed in tumors and that PGE_2 _levels are increased in tumors. COX independent mechanisms for cancer prevention have been shown by Rigas and collaborators [6,7].

Based on the stimulation of AA release by several known cancer preventive agents, e.g., tamoxifen, 9-*cis*-retinoic acid, vitamin D_3_, statins, anti-oxidants found in green tea and red wine, and peroxisome proliferator-activated receptor ligands, I have proposed that AA release by cells is associated with cancer prevention [8-10]. Celecoxib, a COX inhibitor, releases AA from rat liver, human colon carcinoma, and rat glial cells in culture. Three of these cells, rat liver (the C-9 cell line), the human colon cancer (the HT-29 cell line) and rat glia (the C-6 cell line) express COX [6,11,12]. The human colon carcinoma cell line (HCT-15) does not produce prostaglandins (PG) when treated with know stimulators of PG synthesis such as mellitin, A-23187, fetal bovine serum or exogenous AA. Nor do these cells express COX as measured by Northern Blotting (6). In this report, evidence is presented that NSAIDs and other cancer preventive agents release AA from these HCT-15 cells.

Primary cultures of gastric mucosal cells undergo necrosis as well as apoptosis after incubation with NSAIDs [13]. Thus, the effects of NSAIDs on the morphology of sheep erythrocytes (SRBC), cells that also do not express COX [14,15] were also examined using lysis as an indicator of membrane perturbation and necrosis. Some NSAIDs as well as tamoxifen and simvastatin hemolyze the washed COX negative SRBC. Several of the NSAIDs are known to intercalate the plasma membrane of cells [2,3].

## Results

**Figure 1:**
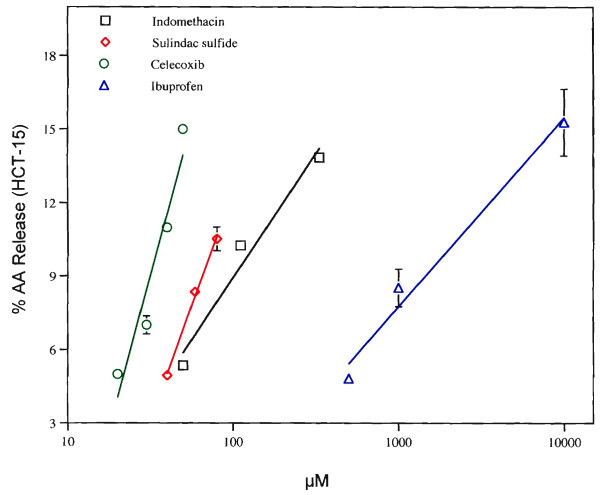
Release of AA from HCT-15 cells by increasing quantities of celecoxib (O), sulindac sulfide (◇), indomethacin (□) or ibuprofen (△). The HCT-15 cells were incubated with the NSAIDs for 19 h. Centrifuged culture fluids (200 μl) were counted for radioactivity.

Preliminary experiments showed that the release of AA from HCT-15 cells by 50 μM celecoxib was complete after a 19 h incubation. The release of AA from these cells by the COX inhibitors, celecoxib (50 μM) and NS398 (52 μM), is shown in Table 1. Celecoxib is more effective than NS398 at comparable doses. The concentration dependencies of the release by celecoxib, sulindac sulfide, indomethacin and ibuprofen are shown in Fig. 1. Celecoxib is 40 to 100 times more effective than ibuprofen, and more effective than sulindac sulfide or indomethacin. In contrast to the observations presented in this report, indomethacin, 200 μM, *inhibited *unstimulated AA release from rabbit kidney slices 50% [16]. At 15 μM, indomethacin *did not inhibit *AA release from perfused rabbit kidney after a 1 h incubation, *but did inhibit its rate of release after *incubation with angiotensin II [16]. In our studies, indomethacin stimulates AA release from unstimulated human colon carcinoma (HCT-29 cells), rat liver (C-9 cells), rat glial (C-6 cells) [8,9,17,18] and human colon carcinoma (HCT-15 cells).

**Figure 2:**
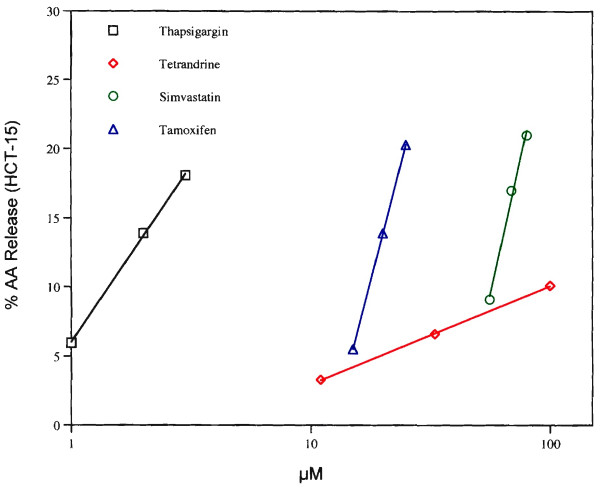
Release of AA from HCT-15 cells by various concentrations of thapsigargin (□), tamoxifen (△), simvastatin (○) and tetrandrine (◇). The experiment was done with triplicate dishes. Incubation conditions were similar to those described in Fig. 1.

The dose-dependencies of AA release by tamoxifen and simvastatin as well as those of thapsigargin and tetrandrine are shown in Fig. 2. Thapsigargin is active even at 1.0 μM. It stimulates AA release from HT-29 cells [18] and induces apoptosis in androgen-independent prostate cancer cell line [19]. Its analogs exhibit chemoprevention of androgen-independent prostate cancer [20]. Thapsigargin also induces apoptosis of human neuroblastoma, colon cancer and thymocytes [21,22]. Tetrandrine stimulates AA release from HT-29 cells [18] and is known to induce apoptosis and is chemopreventive [23]. The dose-response curve of tetrandrine is markedly more shallow than those of the NSAIDs examined and suggests a different mechanism of AA release.

**Table 1:** Release of AA from HCT-15 cells by celecoxib (50 μM) and NS-398 (52 μM). HCT-15 cells, 0.3 to 1.0 × 10^5^/dish, were incubated with the celecoxib, NS-398 or control for 19 h at 37°C. After centrifugation, culture fluids (200 μl) were counted for radioactivity.

Drug tested	cpm/200 μl	AA Release %
None	706	2.7
	947	3.6
(Control MEM/BSA)	943	3.6
	799	3.1
	793	3.0
	m = 838 ± 46.8	m = 3.2 ± 0.18

Celecoxib (50 μM)	3740	14.4
	4441	17.1
	3851	14.8
	4166	16.0
	4413	16.9
	m = 4122 ± 143	m = 15.8 ± 0.54

NS398 (52 μM)	1140	4.4
	1328	5.1
	1223	4.7
	1249	4.8
	1186	4.6
	m = 1225 ± 32	m = 4.7 ± 0.12

Input = 32,880 cpm/200 μl

Cells = 26,045 cpm/200 μl

Uptake = 79%

The insolubility of several of these drugs precludes generation of extensive dose-response curves at high concentrations. At concentrations at which they were soluble, they release a significant amount of AA (Table 2). The concentrations of NSAIDs required for release of AA are greater than those required to inhibit COX as measured by PGI_2 _production of lactacystin plus TPA stimulated COX positive rat liver cells (data not shown). Indomethacin, 50 μM, significantly stimulates AA release from the HCT-15 cells, but 0.003 μM, 4 orders of magnitude less, is required to inhibit 50 % of PGI_2 _produced by different cells (the COX positive C-9 rat liver cells) stimulated by lactacystin plus TPA treatment. COX-inhibition depends on several factors including time of incubation, cellular source of the COX, stimulator of the COX (endotoxin, A-23187, TPA, etc.), subcellular location of the COX, concentration of substrate and the degree of COX-PLA_2 _coupling. For example, the IC_50 _for COX-2 inhibition of PG production by indomethacin when treated with endotoxin-stimulated broken macrophage was 1.1 μM, but when assayed with purified enzyme, it was 14 μM [24].

**Table 2:** Release of AA from HCT-15 cells by NSAIDs and several cancer preventive agents. Such experiments were performed several times with similar results. The experimental conditions are similar to those given in Table 1.

**Drug tested**	Concentration	AA Release *, †
MEM/BSA (control)	0	3.2 ± 0.09 (5)
NaCl	10 mM	3.3 ± 0.12 (3)
A) NSAIDs **		
Acetylsalicylic acid	10 mM	3.6 ± 0.13 (5)
Diflunisac	460 μM	6.7 ± 0.28 (3)
Naproxine	3 mM	4.1 ± 0.29 (3)
Na salicylate	10 mM	5.6 ± 0.18 (5)
NS398	52 μM	4.7 ± 0.35 (5)
Piroxicam	10 mM	4.2 ± 0.33 (5)
Rofecoxib, (Vioxx^®^) ***	100 μM	4.2 ± 0.41 (3)
Valdecoxib, (Bextra^®^)***	178 μM	6.2 ± 0.44 (3)
**B) Drugs that are not NSAIDs – several are cancer preventative**.		
Acetaminophen	10 mM	4.0 ± 0.11 (5)
(-)-Epigallocatechin gallate	224 μM	4.3 ± 0.19 (3)
17 β-Estradiol	100 μM	4.5 ± 0.48 (3)
GW-7845	50 μM	9.5 ± 0.39 (3)
Raloxifene	50 μM	10.9 ± 0.47 (4)
Raloxifene	100 μM	13.3 ± 0.64 (3)
9-*cis*-retinoic acid	83 μM	7.5 ± 0.36 (3)
Resveritrol	100 μM	5.0 ± 0.29 (3)

* = All values except for NaCl, are statistically significant.

** = Solubility of drugs in MEM/BSA does not permit generation of dose-response curves.

*** = DMSO extracts of tablet – 100 % yield assumed.

† = These values are from one experiment. Each drug and controls were examined at least two times. In each experiment these drugs gave statistically significant stimulation *vs *MEM/BSA.

**Figure 3:**
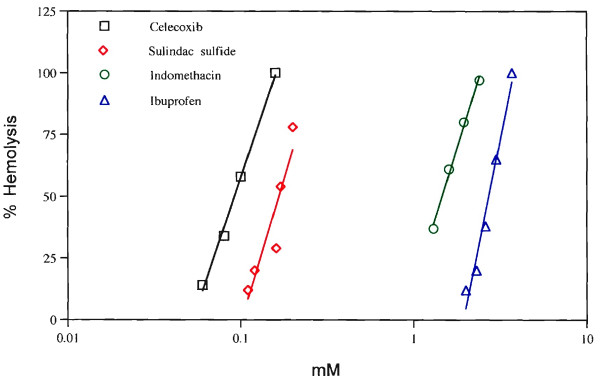
Hemolysis of 6.5 × 10^7 ^SRBC (2.6 × 10^7^/ml) by various concentrations of celecoxib (□), sulindac sulfide (◇), indomethacin (○) and ibuprofen (△). Experimental conditions were similar to those described in Table 4.

**Figure 4:**
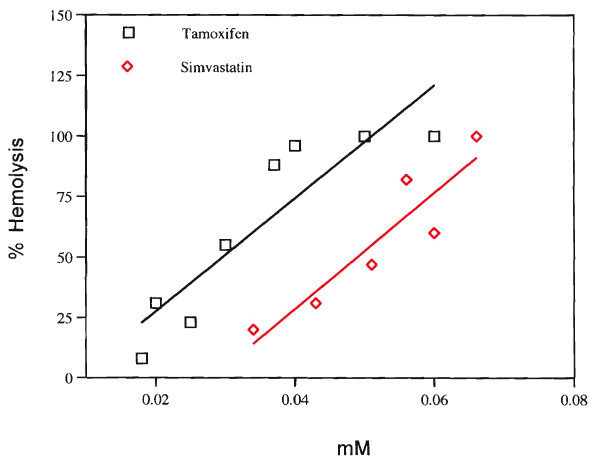
Hemolysis of 6.5 × 10^7 ^SRBC (2.6 × 10^7^/ml) by various concentrations of tamoxifen (□) and simvastatin (◇). Experimental and controls are described in Table 4.

A low concentration of SRBC (6.5 × 10^7 ^cells/ml) was used to quantitate hemolysis [25] after incubation of these COX negative cells with several NSAIDs, tamoxifen or simvastatin. Hemolysis by celecoxib (80 μM) and tamoxifen (20 μM and 40 μM) is shown in Table 3. The concentration dependencies for hemolysis by celecoxib, sulindac sulfide, indomethacin and ibuprofen are shown in Fig. 3. As with AA release (Fig. 1), celecoxib is the most effective. Treatment of cells with tamoxifen and simvastatin also hemolyze the SRBC (Fig. 4). Tetrandrine or thapsigargin, even when tested at 1 mM and 100 μM respectively do not lyse the SRBC (data not shown), suggesting that the mechanisms of AA release by tetrandrine and thapsigargin differs from that of NSAIDs, tamoxifen or simvastatin.

**Table 3:** Hemolysis of 6.5 × 10^7^(2.6 x10^7 ^/ml) SRBC by celecoxib (80 μM) and tamoxifen (20 and 40 μM). The SRBC (2.5 ml) were incubated in a shaking bath for 19 h at 37°C. The cells were centrifuged and the supernates analyzed for oxyhaemoglobin by absorption at 413 nm. Complete hemolysis was calculated from the absorption of the H_2_O lysate of the centrifuged cells of the controls. Controls for absorption at 413 nm of the reagents in the PBS/BSA vehicle as well as controls that contained known amounts of SRBC lysate were used to measure the effects of NSAIDs, if any, on oxyhemoglobin absorption.

Reagent	Hemolysis * %	Absorbance 413 nm
H_2_O	100	2.442
		2.439
		2.423
		2.427
		m = 2.433 ± 0.01

Celecoxib (80 μM)	29	0.799
		0.912
		0.988
		0.635
		m = 0.834 ± 0.077

Tamoxifen (40 μM)	96	2.293
		2.349
		2.396
		2.352
		m = 2.348 ± 0.02

Tamoxifen (20 μM)	29	0.789
		0.844
		1.044
		0.701
		m = 0.845 ± 0.07

PBS/BSA (Control)	0	0.193
		0.214
		0.186
		0.134
		m = 0.182 ± 0.017

* = % Hemolysis of 6.5 × 10^7 ^SRBC (2.6 × 10^7 ^SRBC/ml).

When the effect of cell density on hemolysis was tested with celecoxib, tamoxifen or simvastatin, the number of cells lysed was higher at the lower cell densities (data not shown). Under the experimental conditions described, an approximate number of molecules of drug required to hemolyze 2.5 × 10^7 ^SRBC was calculated (Table 4). These values may be grossly overestimated since binding to the 1 % bovine serum albumin (BSA) in the vehicle was not considered.

**Table 4:** Number of molecules of drug per erythrocyte required for hemolysis of 2.6 × 10^7^SRBC.

Drug	μM required for lysis of 2.6 × 10^7^/ml SRBC.	Molecules per erythrocyte
Tamoxifen	31 μM	7.3 × 10^8^
Simvastatin	49 μM	1.1 × 10^9^
Celecoxib	95 μM	2.2 × 10^9^
Sulindac sulfide	178 μM	4.1 × 10^9^
Indomethacin	1.5 mM	3.5 × 10^10^
Ibuprofen	2.75 mM	6.4 × 10^10^

* = % Hemolysis of 6.5 × 10^7 ^SRBC (2.6 × 10^7 ^SRBC/ml).

## Discussion

In contrast to HT-29 cells which are known to be COX dependent, HCT-15 cells do not respond to known stimulants of COX activity, such as mellitin, A-23187, fetal bovine serum or even exogenous AA [6]. Nor are transcripts of mRNA from both COX-1 and COX-2 genes detected in these HCT-15 cells [6]. When measured by confocal microscopy, however, COX is detected in these HCT-15 cells [26]. A study of AA release by the COX-null embryo fibroblasts described by Zhang et al [27] would be informative and provide another COX negative cell for study. Although erythrocytes, are COX negative [14,15], they may express lipoxygenase (LOX). Sulindac sulfide and indomethacin stimulate AA release from the colorectal cancer cell lines, SW480 and HCT-116. It is the AA release *per se *that leads to apoptosis [28]. Chan *et al *[28] have suggested that inhibition of COX is a mechanism of action of NSAIDs. By inhibiting COX, more AA is made available as substrate for sphingomyelin to ceramide conversion. I have proposed that the release of AA is associated with cancer prevention [8-10,17,18]. The AA release was observed from COX positive rat liver (C-9), COX positive human colon cancer (HT-29) and COX positive rat glioma (C-6) cells. Growth of COX negative and COX positive cells is inhibited and their progression to apoptosis is increased by NSAIDs [6,7]. In cells different than those cited above, others have demonstrated COX independence for NSAID actions [reviewed in [29,30]].

**Figure 5:**
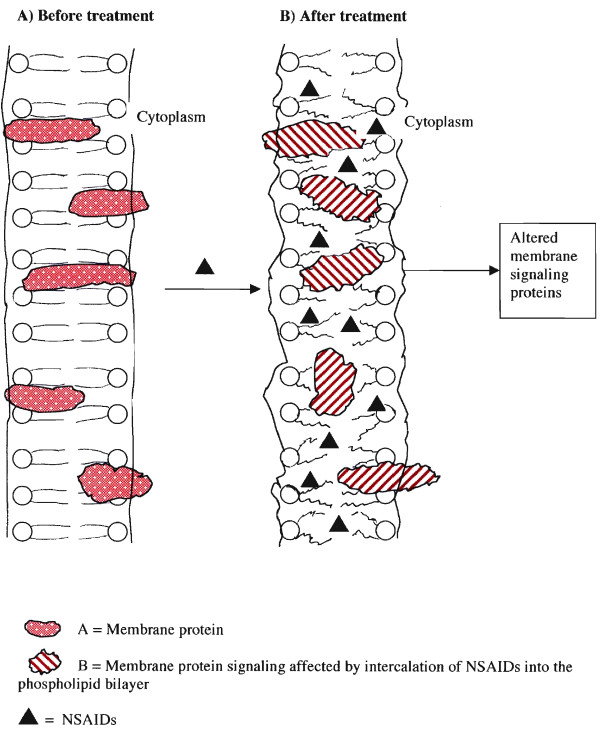
Schematic representation of the mechanism of action of high doses of NSAIDs on cell membranes.

Red blood cells do not express COX activity [14,15], yet incorporate AA into their phospholipids. After a 5 min incubation with the Ca^2+ ^ionophore, A-23187, 26 % the AA is found unesterified when analyzed by high pressure liquid and thin layer chromatography [14]. In the present study, hemolysis is observed after a 19 h incubation of SRBC with NSAIDs, tamoxifen or simvastatin. Celecoxib is about 30 times more effective than ibuprofen (Fig. 3 and Table 4). The relative activities of celecoxib, rofecoxib and valdecoxib for the necrotic stage of the actions of NSAIDs, as measured by hemolysis of SRBC, may be associated with an unwanted side reaction. The hemolysis suggests a mechanism proposed much earlier [2,3] for the effects of NSAIDs on a variety of biological pathways, namely, perturbation of the cell membranes and disruption of normal signaling pathways (Fig. 5). The deesterification of phospholipids could lead to altered signaling. It is not restricted to NSAIDs. Even at high concentrations, AA release is stimulated by tamoxifen. It is independent of estrogen synthesis [9]. The stimulation of AA release by simvastatin is independent of cholesterol synthesis [10]. Simvastatin also hemolyzes SRBC (Fig. 4). The significant correlation between high doses of statins and bone fractures [31] may reflect intercolated cell membranes of cells relevant to bone integrity, i.e. the biological response to high doses of such drugs may depend on the function of the target cell.

COX inhibitors leading to decreased AA metabolism may be the primary pathway for combating pain. The perturbation of the membrane and the accompanying changes in signaling may be responsible for many of their other biological effects, including cancer chemoprevention, reduction of amyloid production in Alzheimer disease [32] and possibly even production of lesions in the gastric mucosa [33]. It would be of interest to assess epidemiologically other conditions that may be affected by exposure to high doses of NSAIDs, e.g., cardiotoxicity. The effects of these agents do not appear to be selective to cancer cells.

The numerous findings of overexpression of COX-2 and elevated PGE_2 _levels in cancer as well as the genetic evidence [reviewed in [29,30]] have lead to the concept that COX-2 is a primary target for cancer prevention. Alternatively, the effects on cancer prevention observed with NSAIDs and some other agents may result from the stimulation of AA release followed by the altered downstream signaling. Lypooxygenase (LOX) and LOX products (5-LOX and 12-LOX) are up-regulated in human pancreatic cancer. Inhibitors of LOX induce apoptosis in these human pancreatic cell lines [reviewed in [29]]. Since LOX activity was not examined in the HCT-15 cells, the possibility that LOX is a target for the NSAIDs cannot be ruled out. LOX activity is present in the SRBC undergoing hemolysis but the effect of NSAIDs on the production of LOX products was not studied.

The majority of the compounds tested for release of AA from cells in culture are effective at concentrations of 50 μM or greater. Such concentrations are significantly higher than those found in patients receiving i.e., indomethacin, 20 μM, based on serum levels. However, based *only *on serum levels, estimations of ligand concentration effective on the target cells can be misleading. For example, sulindac sulfide levels may be 20-fold higher in colonic epithelial cells of guinea pigs than in serum [34]. In addition, comparison of the effects of drug concentrations in cell cultures and in humans is subject to inherent experimental differences. High doses of NSAIDs are used in cell culture experiments while low doses may be effective in man. In cell culture experiments the time of incubation of cells with NSAIDs is of necessity relatively short whereas in patients, the effects are usually observed after much longer periods of time of exposure, sometimes years. The physiological relevance of the high levels of NSAIDs found *in vitro *has been addressed in recent reviews [29,30].

## Conclusion

A cell that does not express COX activity (HCT-15) releases AA when incubated with NSAIDs and cancer preventive agents. COX negative SRBC also undergo lysis when incubated with NSAIDs, tamoxifen or simvastatin suggesting that the mechanism of action of these drugs involves their intercalation of the membrane and disruption of signaling messages.

## Methods

The human colon carcinoma (HCT-15 cell line) and the rat liver (C-9 cell line) were purchased from the American Type Culture Collection (Manassas, VA, USA). They were maintained in Eagle's minimum essential medium (MEM) supplemented with 10% fetal bovine serum. [^3^H] AA (91.8 Ci/mmol) was obtained from NEN Life Science Products, Inc. (Boston, MA, USA). The washed SRBC were purchased from Lampire Biological Laboratories (Pipersville, PA, USA). All other reagents were from Sigma Chemical Co. (St. Louis, MO, USA) or Calbiochem, (San Diego, CA, USA).

Two days prior to experiments, the HCT-15 or C-9 cells were treated with 0.25% trypsin-EDTA and, after addition of minimal essential media (MEM) containing 10% fetal calf serum, the floating cells were seeded on to 35 mm culture dishes. The plating densities varied from 0.1 to 0.5 × 10^5 ^cells/35 mm dish. The freshly seeded cultures were incubated for 24 h to allow for cell attachment. After decantation of MEM containing the fetal bovine serum, 1.0 ml fresh MEM containing 10% fetal bovine serum and [^3^H] AA (0.2 μCi/ml) was added and the cells incubated for another 24 h. The cells were washed 4 times with MEM and incubated for 19 h with 1.0 ml of MEM containing 1.0 mg BSA/ml (MEM/BSA) and different concentrations of each compound. The culture fluids were then decanted, centrifuged at 2000 x g for 10 min, and 200 μl of the supernate counted for radioactivity. Radioactivity recovered in the washes before the incubation was compared to input radioactivity to calculate the % radioactivity incorporated into the cells. The MEM/BSA values are the control values. The [^3^H] AA release is presented as a percentage of the radioactivity incorporated by the cells. In 56 experiments, the average release of AA from HCT-15 cells in the presence of MEM/BSA was 3.2 ± 38 %. For all data presented, values are normalized to an MEM/BSA value of 3.2%. Three to five culture dishes were used for each experimental point. The data are expressed as mean values ± SEM. The data were evaluated statistically by the unpaired *Student's t-test*. A *P *value < 0.05 was considered significant.

For measuring the effect of NSAIDs on PGI_2 _production, the C-9 cells were incubated for an additional 24 h with MEM plus 10% fetal bovine serum (minus [^3^H] AA). The cells were washed three times with MEM and incubated with 5.4 μM lactacystin plus 17 mM TPA in MEM/BSA in the presence of various concentrations of NSAIDs for 6 h. The culture fluids were centrifuged and the supernates analyzed for 6-keto-PGF_1α_, the stable hydrolytic product of PGI_2_, by radioimmunoassay [35].

For the hemolysis studies, 100 μl of the SRBC suspension were washed twice with about 15 ml of phosphate buffered saline (pH 7.3) containing BSA (1 mg/ml) (PBS/BSA). The washed cells were resuspended in 50 ml PBS/BSA and 2.5 ml (2.6 × 10^7^/ml) were incubated with varying concentrations of the reagents for 19 h at 37°C. The reaction mixtures were centrifuged and the oxyhaemoglobin measured by absorption at 413 nm. The pellets containing the intact erythrocytes were lysed with 2.5 ml H_2_O and they too measured for absorption. To determine the number of cells in the experiment, 1 ml of the SRBC suspension was diluted to 15 ml with H_2_O and the lysate measured for absorption. Controls were performed by incubation of each test reagent in the vehicle alone and measured for absorption in the presence and absence of oxyhaemoglobin. Only sulindac sulfide and indomethacin, at the concentrations used, absorbed at 413 nm. Ibuprofen, at concentrations above 3.5 mM, reduced oxyhaemoglobin absorption.
